# Strengthened structure–function relationships of the corticospinal tract by free water correction after stroke

**DOI:** 10.1093/braincomms/fcab034

**Published:** 2021-04-28

**Authors:** Stephanie Guder, Ofer Pasternak, Christian Gerloff, Robert Schulz

**Affiliations:** 1 Department of Neurology, University Medical Centre Hamburg-Eppendorf, 20246 Hamburg, Germany; 2 Departments of Psychiatry and Radiology, Brigham and Women's Hospital, Harvard Medical School, Boston, MA 02115, USA

**Keywords:** TMS, MEP, recruitment, CST, excitability

## Abstract

The corticospinal tract is the most intensively investigated tract of the human motor system in stroke rehabilitative research. Diffusion-tensor-imaging gives insights into its microstructure, and transcranial magnetic stimulation assesses its excitability. Previous data on the interrelationship between both measures are contradictory. Correlative or predictive models which associate them with motor outcome are incomplete. Free water correction has been developed to enhance diffusion-tensor-imaging by eliminating partial volume with extracellular water, which could improve capturing stroke-related microstructural alterations, thereby also improving structure-function relationships in clinical cohorts. In the present cross-sectional study, data of 18 chronic stroke patients and 17 healthy controls, taken from a previous study on cortico-cerebellar motor tracts, were re-analysed: The data included diffusion-tensor-imaging data quantifying corticospinal tract microstructure with and without free water correction, transcranial magnetic stimulation data assessing recruitment curve properties of motor evoked potentials and detailed clinical data. Linear regression modelling was used to interrelate corticospinal tract microstructure, recruitment curves properties and clinical scores. The main finding of the present study was that free water correction substantially strengthens structure-function associations in stroke patients: Specifically, our data evidenced a significant association between fractional anisotropy of the ipsilesional corticospinal tract and its excitability (*P* = 0.001, adj. *R*^2^ = 0.54), with free water correction explaining additional 20% in recruitment curve variability. For clinical scores, only free water correction leads to the reliable detection of significant correlations between ipsilesional corticospinal tract fractional anisotropy and residual grip (*P* = 0.001, adj. *R*^2^ = 0.70) and pinch force (*P* < 0.001, adj. *R*^2^ = 0.72). Finally, multimodal models can be improved by free water correction as well. This study evidences that corticospinal tract microstructure directly relates to its excitability in stroke patients. It also shows that unexplained variance in motor outcome is considerably reduced by free water correction arguing that it might serve as a powerful tool to improve existing models of structure-function associations and potentially also outcome prediction after stroke.

## Teaser

Guder et al. investigated FWC of diffusion-weighted imaging data in chronic stroke patients. Showing that this approach can strengthen the relationship between microstructural properties of the cortico-spinal tract, measures of cortical excitability and also behaviour suggests its potential to improve our existing models of structure-function associations after stroke.

## Graphical Abstract

**Graphical Abstract:**
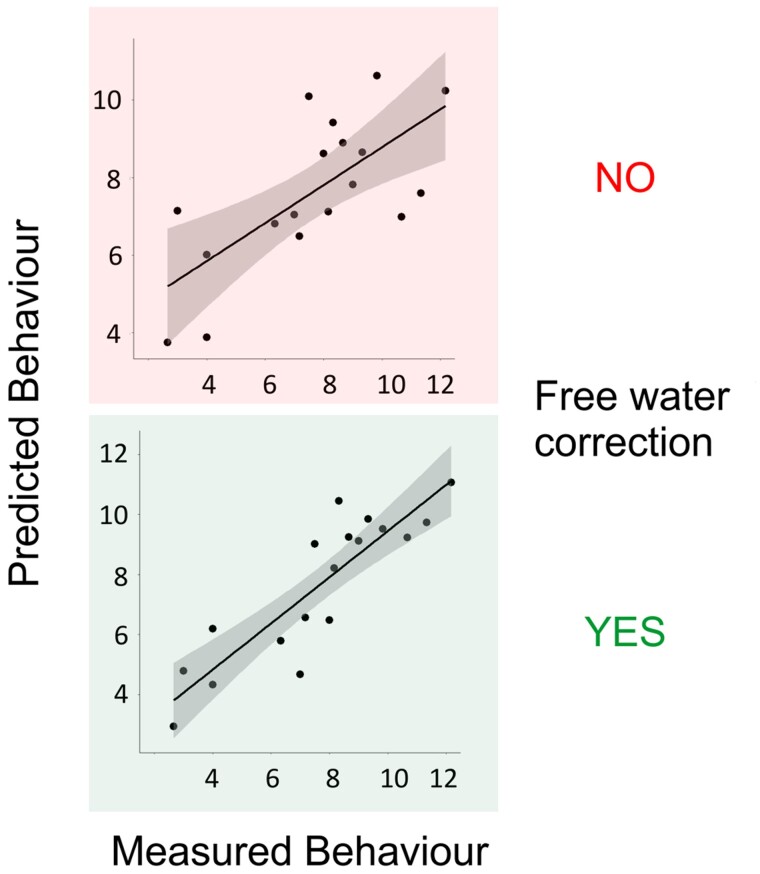

## Introduction

Systems neuroscience and neurorehabilitation research have continuously developed powerful tools from diffusion MRI to investigate macro- and microstructural changes after stroke and to understand how altered brain tissue and brain networks influence recovery processes. A variety of studies has focused on the corticospinal tract (CST), the main outflow tract of the human motor system.^1^ Aside from mere lesion load,^2^ the microstructure of the ipsilesional CST, most widely assessed by means of the diffusion-tensor-imaging (DTI) derived parameter fractional anisotropy (FA), has been repeatedly shown to contribute significantly to associative and predictive models of structure-behaviour relationships.^1^ However, a recent meta-analysis has indicated high variability in the amount of explained variance in regression analyses between FA of the CST and motor function or subsequent recovery.^3^ Likewise, also the relationship between the amount of damage to the CST and its electrophysiological excitability, measured by means of transcranial magnetic stimulation (TMS), shows a high degree of inter-subject variability. Results on the interrelationship between CST structure and function are contradictory. There is only one study that showed a significant association between excitability as assessed by means of mere active motor threshold and gross lesion load to the ipsilesional CST.^4^ With regard to CST microstructure or the count of residual fibre tracts, other studies failed to detect significant associations between recruitment curve (RC) properties of motor-evoked potentials (MEPs) and damage to the ipsilesional CST.^5^^,^^6^ To explain inconsistencies between studies, it has been hypothesized that only fibres emerging from the primary motor cortex (M1) might inform about excitability alterations after stroke. These fibres might have not been sufficiently assessed by the imaging techniques applied. Though in fact, one study that investigated different subcomponents of the CST originating from M1 and also secondary motor areas has failed to detect any significant associations between RC characteristics and the FA of M1-related corticospinal motor fibres.^7^

Given the intuitively expected interrelationship between alterations in CST microstructure and CST excitability in stroke patients,^4^^,^^8^ the question arises whether further improvement of FA estimation of the CST might help to strengthen such structure-function associations, ultimately also to enhance correlative and predictive models in neurorehabilitation research. Contamination of diffusion measures of the CST by free water (FW)^9^ has been increasingly recognized as a relevant issue in structural brain imaging. Previous data have shown that increased FW content is not only detectable in brain tissue directly affected by the stroke lesion but also distant from it in the cerebral peduncles.^10^ TMS measures of CST excitability are unlikely to be influenced by FW content. This might explain why previous studies failed to uncover robust relationships between CST structure and CST function in stroke patients. Hence, FW correction (FWC) might serve as one option to come closer to the ground truth of alterations of CST microstructure. Fitting bi-tensor models allow to separate diffusion properties of brain tissue from surrounding FW in order to correct diffusion-data for atrophy-based partial volume FW contamination or water uptake due to vasogenic oedema in stroke lesions.^9^^,^^11^ So far, there are only few studies exploring the potential of bi-tensor models to account to FW. These studies could relate FW itself and FA of the CST after FWC to residual motor output^10^ and found that such models provided stronger relationships with impairment compared to uncorrected diffusion measures.^12^ However, the demonstration of an improved specificity of corrected CST measures to its excitability properties is not available.

The aim of this study was to test the hypothesis that FW corrected DTI-based microstructural properties of the CST will have a stronger association with its excitability as assessed by means of TMS and also with residual motor output after stroke. We therefore re-analysed available diffusion data from eighteen chronic stroke patients from a previous report on the influence of cortico-cerebellar motor pathways to cortical excitability after stroke.^13^ Here, we applied novel statistical modelling to correlate tract-related diffusion-metrics *with* and *without* FWC with RC properties of MEPs and behavioural measures. Importantly, the RC data and behavioural results are taken from the previous report 1:1 and will be only summarized in adapted forms to ensure an understanding of the current protocols.

## Materials and methods

### Participants

Demographic and clinical data are introduced in detail in our previous report.^13^ In brief, eighteen chronic patients (aged 66.7 ± 2.2 years, mean ± SEM, range 53–84, 15 males, one left-handed) with supratentorial ischemic stroke lesions and persistent motor deficits of the upper extremity were included in this cross-sectional study. Patients gave written informed consent according to the Declaration of Helsinki to participate in the study, which was approved by the local ethics committee (PV5357). Clinical testing included grip force, pinch force and the nine-hole-peg-test (in pegs/seconds) and the Fugl-Meyer assessment of the upper extremity (UEFM). Seventeen healthy participants (aged 66.8 ± 2.0 years, range 55–79, 14 males, one left-handed) were also recruited. Controls were pseudo-randomly assigned to be treated as ‘dominant hemisphere affected’ (*n* = 13); brain imaging and TMS experiments and analyses were conducted accordingly to account for the distribution of lesions to the dominant and non-dominant hemispheres in the stroke patients. Table 1 summarizes clinical data of the stroke patients, for controls, the demographic and clinical data are summarized in Supplementary Table 1.

**Table 1:** Demographic and clinical data of the stroke patients

ID	Gender	Age	DoHe	AfHe	Time	Stroke	Grip force		Pinch force		NHP		UEFM
							AH	UH	AH	UH	AH	UH	
1	M	77	L	L	28	BG, IC	36.3	40.3	0.4	0.6	11.3	7.8	60
2	F	78	L	L	20	BG, IC	19.3	24.0	0.8	0.8	7.0	7.5	66
3	M	74	L	L	20	CR	42.0	34.7	0.6	0.5	10.7	8.8	66
4	F	73	L	R	26	MCA	9.7	19.3	0.3	0.7	2.7	4.7	52
5	M	61	L	R	35	MCA	28.7	45.3	0.7	0.7	9.3	9.5	66
6	M	55	L	L	66	BG, IC	46.3	45.3	0.7	0.9	12.2	11.3	66
7	M	75	L	R	58	PLIC	26.7	40.7	0.3	0.8	7.5	12.0	39
8	M	61	L	L	75	BG, IC	31.0	42.7	0.5	0.8	8.0	9.8	47
9	M	53	L	L	45	PG	37.7	37.3	0.9	0.9	8.7	9.7	66
10	M	76	L	L	80	BG, IC	24.3	31.7	0.4	0.8	8.2	8.5	50
11	M	61	L	L	88	PLIC	31.0	34.7	0.7	0.7	8.3	9.7	64
12	M	73	R	R	62	BG, IC	31.3	35.3	0.6	0.7	9.8	10.7	63
13	M	60	L	L	9	TC	35.3	43.3	0.6	0.7	9.0	8.2	55
14	M	58	L	L	31	TC	24.0	22.3	0.9	0.9	7.2	6.2	66
15	M	64	L	R	7	BG, IC	4.3	21.3	0.5	0.8	4.0	6.7	52
16	M	63	L	L	11	BG, CR	20.3	46.0	0.5	1.0	3.0	8.0	51
17	F	84	L	L	23	BG, CR	9.3	15.0	0.4	0.7	4.0	6.3	39
18	M	54	L	L	29	CR, PLIC	17.3	42.0	0.6	0.7	6.3	8.8	59
Mean stroke	M : 15	66.7	L : 17	L : 13	39.6	–	26.4^*^	34.5	0.6^#^	0.8	7.6^*^	8.6	57.1
SEM stroke	–	±2.2	–	–	±6.0	–	±2.7	±2.4	±0.04	±0.03	±0.7	±0.4	±2.2

Gender (M = male; F = female) and age (in years), dominant (DoHe) and affected (AfHe) hemisphere (L = left; R = right), Time time after stroke (in months), stroke (location, TC = thalamocapsular; CR = corona radiata; IC = capsula interna; PLIC = posterior limb of the internal capsule; BG = basal ganglia; PG = precentral gyrus; MCA = extended media infarct), Absolute pinch and grip force values (both in kg). NHP = Nine-hole-peg performance (in pegs per seconds; AH = affected hand; UH = unaffected hand). UEFM (Fugl-Meyer assessment of the upper extremity). SEM standard error of the mean. *Indicates significant difference between AH and UH in patients with *P* < 0.05, ^#^* *= 0.08, for details see Guder et al.^13^

### TMS data acquisition and analysis

**Figure 1:**
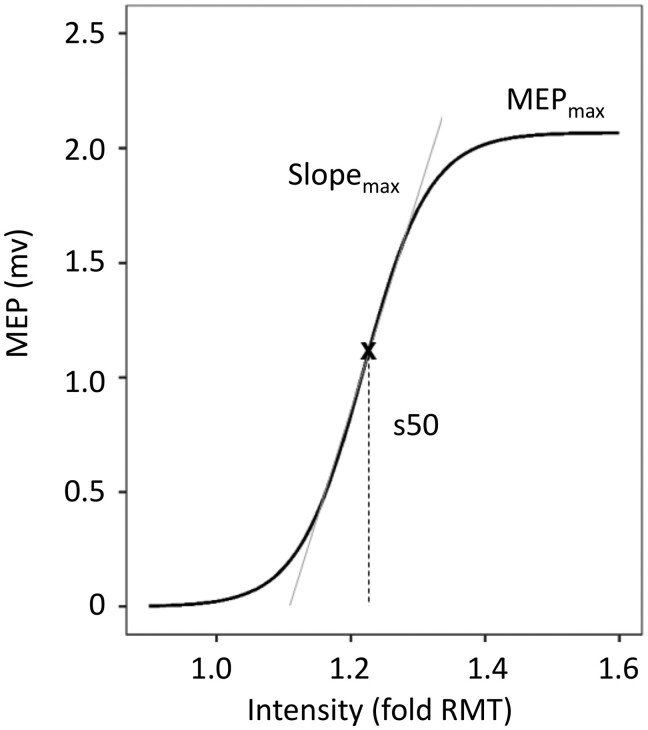
**Example of fitted recruitment curve using the Boltzmann Equation.** Recruitment curves of MEPs acquired by TMS were fitted using the Boltzmann Equation. Parameters of the curve (MEPmax, Slope_max_, s50) were obtained for further analysis (adapted from Guder et al.^13^).

TMS data are taken from our previous report.^13^ To summarize, data acquisition was conducted using a Magstim 200 magnetic stimulator with a figure of eight coil with a 70 mm wing diameter and EMG electrodes placed over the first dorsal interosseous muscle on both hands in a belly-tendon montage. Methods are described in detail in our previous report.^13^ In brief, at the MEP hot spot over M1, the resting motor threshold (RMT) was determined to the nearest 1% of the maximum stimulator output (MSO). To obtain properties of the MEP RC,^14^ we used blocks of 11 stimuli for each intensity value, ranging from 90 to 160% of RMT. The first trial in each block was discarded. The order of the stimuli intensities was pseudo-randomized in order to avoid hysteresis effects. RCs were measured at both hemispheres. Data analysis was conducted using Signal software 4.05 (Cambridge Electronic Design, Cambridge, UK). Fitting of RC was based on at least 5 trials per stimulus intensity from 90 to 160% RMT except for one patient and one control participants in which 160% RMT could not be reached due to high RMT and increased stimulation intensities at 160% (>90% of total MSO). R Studio Version 1.1.383 (http://www.r-project.org/) was used to fit the three parameter sigmoid Boltzman function (Equation 1) to each individual RC, thereby estimating MEPmax (plateau of RC), SLOPEmax (slope of RC, straight line fitted to RC at its inflection point) and stimulus intensity s50 to obtain a response 50% of the maximum (Fig. 1).

(1) $$y=\frac{\mathrm{MEPmax}}{{1\;+\;e}^{\frac{s50-s}{k}}}\;$$

Table 2 summarizes the relevant TMS values used in this study. There was a significant reduction in MEPmax and SLOPEmax of the affected hemisphere (AH). For further details, we refer the reader to the original publication.^13^

**Table 2:** Characteristics of cortical excitability

	MEPmax^#^		SLOPEmax^#^		s50	
	AH	UH	AH	UH	AH	UH
Stroke	1.6 ± 0.3***	4.0 ± 0.5	4.4 ± 0.9**	9.5 ± 1.3	1.25 ± 0.02	1.32 ± 0.03
Control	3.4 ± 0.4	3.9 ± 0.5	9.6 ± 1.2	10.3 ± 1.4	1.28 ± 0.6	1.29 ± 0.02

Maximum MEP (MEPmax) (in mV), SLOPEmax (max. slope of RC), s50 (inflection point) of the affected (AH) and unaffected (UH) hemispheres. Linear mixed-effects modelling revealed a significant effect of GROUP*SIDE for MEPmax and SLOPEmax. Group comparisons include mean ± standard error of the mean (SEM), and *P*-values (unpaired *t*-tests). Significant group differences are indicated by asterisks (*P <* *0.05, **P <* *0.01, ***P <* *0.001, uncorrected). ^#^Log10 transformed values were used for statistical analysis. Adapted from Guder et al.^13^

### Brain imaging

Available MRI data included high-resolution T1-weighted anatomical and diffusion-weighted images, acquired on a 3-T Siemens Skyra scanner (Siemens, Erlangen, Germany) and a 32-channel head coil. Diffusion-weighted images consisted of 75 axial slices covering the whole brain with gradients (*b* = 1500 s/mm^2^) applied along 64 non-collinear directions and one b_0_ image (TR 10 s, TE 82 ms, resolution 2 × 2 × 2 mm). Brain imaging was conducted using the FSL software package 5.0.2.2 (http://www.fmrib.ox.ac.uk/fsl). After eddy current correction and brain extraction, 4 D volumes were used as inputs in *two different* procedures: (1) DTIFIT, implemented in FSL, was used to calculate FA maps by fitting the diffusion tensor model at each voxel.^15^ Based on the tensor information, maps for alternative diffusion metrics that are mean axial (AD) and radial diffusivity (RD) were also calculated. (2) A custom written MATLAB script (ran on R2016a, The Mathworks, Natick, MA, USA)^9^ was used to estimate FW maps and FA maps, AD and RD maps after FWC. In brief, a bi-tensor model is calculated to predict signal attenuation in the presence of FW contamination. The model includes two different compartments: The first compartment estimates the fractional volume of FW, which is modelled as an isotropic tensor with a fixed diffusivity. The other compartment uses a diffusion-tensor to model water molecules in the vicinity of tissue membranes, from which FWC DTI measures are calculated.^9^ The uncorrected individual FA-maps were subsequently registered non-linearly by means of FSL *flirt* and *fnirt* to the Montreal Neurological Institute (MNI) standard space. The resulting transformation was applied to all other diffusion metrics with and without FWC. To quantify CST microstructure, we used available binarized tract templates to read out mean FA, AD and RD values at the level of the mesencephalon to the cerebral peduncles (CP) from *z* = −25 to −20 in MNI space. Details are given in the Supplementary material and our previous reports.^16^^,^^17^

### Statistics

For statistical analysis, R Studio was used. Statistical significance was assumed at *P*-values ≤ 0.05. A thorough statistical analysis of the TMS parameters or the clinical data was not in the scope of this study, we ask the reader to consult the original report.^13^

For group comparison of CST microstructure (dependent variable) without and with FWC between patients and controls, linear mixed-effects modelling with repeated measures (R’s *lmer*, with factor *SUBJECT* as random term, *GROUP* (stroke patients, controls), *SIDE* (affected [AH] or unaffected [UH] hemisphere) and *FWC* (with or without) as fixed effects) was used. *AGE* was additionally considered as a covariate. Such models were calculated for FA, AD and RD data. Tukey method in R’s *lsmeans* was used for *P*-value adjustment during *post hoc* comparisons of estimates. Estimated means and 95% confidence intervals (CI) are calculated. For FW, one separate LMER model was calculated with the fixed factors *GROUP*, *SIDE*, the covariate *AGE* and *SUBJECT* as random term.

In order to explore the relationship between CST microstructure and TMS parameters of CST excitability and behaviour, we computed individual linear regression models (*lm*). Dependent variables were *TMS parameters* (MEPmax, SLOPEmax and s50) of AH or *behavioural scores* (grip force, pinch force, NHP of AH, UEFM). The independent variable of interest was CST (FA, AD, and RD) with two separate models each for AH and UH and for each outcome measure. Coefficient estimates of CST AH diffusion measure were reported for each model with their *P*-values (within model). *AGE* was included as a covariate. Also, TMS parameters and behavioural scores of the UH were included in the models to adjust the target effects, in line with our previous report.^13^ Model fit without FWC (models 1) was compared to models with FWC (models 2) by evaluation of adjusted *R*^2^, root mean squared error (RMSE) and likelihood-ratio (LR) test. For the LR, we compared models 1 with the according models 2 as nested models to meet test assumptions (i.e. outcome ∼ uncorrected CST FA AH + AGE + corrected CST FA AH). Separate models were fitted to assess the relationship between CST FW and the outcome measures. Variation inflation factor (VIF) analysis was used to exclude multi-collinearity in the models. FDR correction was applied to correct for multiple comparisons.

Finally, in order to evaluate the potential of FWC for multimodal models including CST microstructure and TMS parameters to infer motor outcome, we constructed *lm* models with multiple predictors (details given in the relevant section) and used stepwise backward model simplification to obtain the final models.

### Data availability

Data are available from the corresponding author on reasonable request.

## Results

### Free water correction and CST microstructure

LMER for CST FA did not reveal a significant triple interaction GROUP*FWC*SIDE (*F*_1,105_ = 1.4, *P* = 0.24). However, GROUP*SIDE (*F*_1,105_ = 18.8, *P* < 0.0001) was significant. *Posthoc* comparisons showed that stroke patients exhibit a significantly lower FA in the AH (estimated mean 0.56) compared to UH [0.62], independent from FW elimination (*P* < 0.0001, Table 3). Also, we found that FA UH in stroke patients was lower compared to the FA in controls (0.67, *P* = 0.005). Also GROUP*FWC (*F*_1,105_ = 35.9, *P* < 0.0001) was significant. *Posthoc* comparisons showed that stroke patients had significantly lower FA in both hemispheres than controls after FWC (*P* < 0.0001; FWC-FA stroke = 0.59; FWC-FA controls = 0.72), but not before FWC (*P* = 0.10; FA stroke = 0.59; FA controls = 0.63, all est. means).

**Table 3:** Corticospinal tract microstructural properties with and without free water correction

	Measure	Stroke patients			Healthy controls		
		Est. mean	95% CI		Est. mean	95% CI	
			Lower	Upper		Lower	Upper
No FWC	CST FA AH	0.57	0.53	0.60	0.63	0.59	0.67
	CST FA UH	0.61	0.57	0.65	0.62	0.58	0.66
FWC	CST FA AH	0.55	0.51	0.59	0.72	0.68	0.76
	CST FA UH	0.62	0.58	0.66	0.71	0.67	0.75
	CST FW AH	0.25	0.23	0.27	0.20	0.18	0.22
	CST FW UH	0.21	0.19	0.23	0.22	0.20	0.24

Lmer for CST FW values revealed a significant interaction for GROUP*SIDE (*F*_1,35_ = 12.9, *P* = 0.001) with *posthoc* tests showing that ipsilesional FW in stroke patients (0.25) was significantly higher than their UH (0.21, *P* = 0.01) and compared to controls AH as well (0.20, *P* = 0.001). Notably, FW of CST of both hemispheres significantly increased with age in stroke patients and healthy controls. (*F*_1,35_=25.1, *P* < 0.001). Table 3 gives estimated means for FA and FW irrespectively from non-significant interaction terms. Results for AD and RD values are described in the Supplementary material.

Estimated means with 95% confidence intervals (CI) are given for diffusion properties of the CST of the affected (AH) and unaffected (UH) hemispheres. FWC, FW fractional volume. For group comparisons, please refer to the text. Please note that triple interactions were not significant (see text). Hence, statistical comparisons on these individual values were not conducted. Non-FWC FA values have been already reported in our previous report.^13^

### Association between CST microstructure and cortical excitability

**Figure 2:**
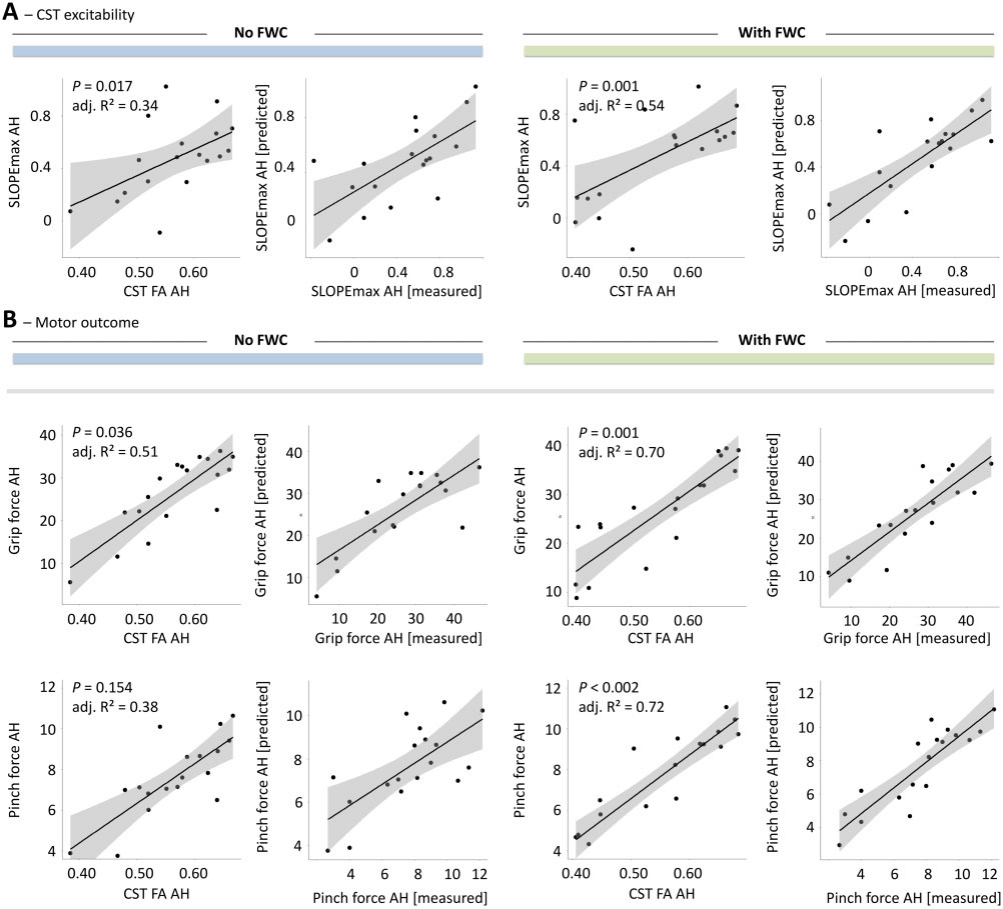
**Association between tract-related microstructure of the corticospinal tract and measures of cortical excitability and behavioural scores.** Effect plots are shown for mean CST FA of the affected hemisphere (AH) contributing to the explanation of CST excitability (SLOPEmax AH) in **A** and motor outcome in **B** (grip force and pinch force of the affected hand (AH)). Left 2 columns: Models without FWC to estimate diffusion properties, right 2 columns: Models with FWC. *P*-value of the tract (within-model) are given (uncorrected). *Raw* tract-related FA values are plotted against *estimated* means of the outcome variable. Adj. *R*^2^ explained variance of the overall regression models with plotting *measured* outcome vs. *predicted* outcome is also given.

After evaluating overall model significances and correcting *P*-values of within-model predictors of interest for multiple comparisons, we found that the mean FWC-FA from the CST of AH was positively associated with maximum RC slope in stroke patients (*P* = 0.001, Table 4, Fig. 2A). Without FWC, the association between FA and RC did not reach statistical significance after FDR correction for 18 tests (*P* = 0.017, uncorrected). LR-test indicated that FWC significantly improved the statistical model with CST FA AH to explain SLOPEmax (LR-test *P* = 0.003, adj. *R*^2^_nested_ = 0.56). In line, FWC added 20% in explained variance to the model and improved overall strength of model fit by 17% in RMSE. For illustration, Fig. 2A plots *measured* vs. *predicted* SLOPEmax for CST FA AH with and without FWC. We have to point out clearly that the non-FWC finding has been already reported as a secondary finding in the original dataset based on a multivariate regression analysis focusing on the importance of cortico-cerebellar fibre tracts. In these analyses though, proportional CST FA served as a covariate to adjust the target effects.^13^

**Table 4:** Associations between tract-related microstructure of the corticospinal tract and measures of cortical excitability

Outcome	Predictor	Model 1 | No FWC			Model 2 | FWC		
		Coef.	*P*	Adj. *R*²	Coef.	*P*	Adj. *R*²
MEPmax AH	CST FA AH	2.25	0.108	0.11	1.96	0.039	0.21
	CST FA UH	−1.25	0.583	−0.06	2.23	0.197	0.04
	CST FW AH	–	–	–	−4.07	0.213	0.04
	CST FW UH	–	–	–	0.13	0.967	−0.08
SLOPEmax AH	CST FA AH	3.37	**0.017^#^**	0.34	2.97	0.001*^r^	0.54
	CST FA UH	−1.51	0.524	0.02	3.00	0.098	0.18
	CST FW AH	–	–	–	−4.53	0.176	0.12
	CST FW UH	–	–	–	1.78	0.597	0.02
s50 AH	CST FA AH	−0.10	0.711	0.33	−0.05	0.800	0.33
	CST FA UH	0.30	0.459	0.35	0.25	0.406	0.36
	CST FW AH	–	–	–	0.84	0.161	0.42
	CST FW UH	–	–	–	−1.20	**0.018^#^**	0.55

Coefficients (Coef.) are given incl. *P*-values of tract of interest (within regression model) in individual models for the 3 outcome variables (dependent variable) and CST FA of the AH or unaffected hemisphere (UH). Middle column: Model 1 without FWC (i.e. only uncorrected FA values), right column: Model 2 with FWC (i.e. only uncorrected FA values) applied to asses CST microstructure. FW values were also used in separate models. Adj. *R*^2^ are given for the complete final models. For model specifications please see the statistics section. *Indicates significant predictors after FDR correction of all *P*-values for multiple testing for 18 test. ^#^Indicates significant predictors based on uncorrected *P*-values in models which reached overall significance. Adjusted (Adj). *R*^2^ of baseline models (e.g. MEPmax AH ∼ MEPmax UH + age) were –0.01 for MEPmax AH, 0.06 for SLOPEmax AH and 0.37 for s50 AH. ^r^FWC leads to a reduction of RMSE by 17% from 0.35 to 0.29. LR-based model comparison reveals a significant model improvement under FWC. For AD and RD values, please see Supplementary Table 3.

There were no significant associations between TMS parameters of cortical excitability and AD or RD values of CST (Supplementary Table 2). The analyses in the healthy controls did not reveal any significant associations between CST microstructure and the TMS parameters of cortical excitability (data not shown).

### Free water correction for CST microstructure and motor outcome

Based on uncorrected statistical results, there were significant associations between CST FA AH and grip force (*P* = 0.036). FWC did reveal significant associations between corrected CST FA AH and both grip force (*P* = 0.001) and pinch force (*P* < 0.001) and between CST FW values and NHP and UEFM (Table 5). However, the only regressions which remained significant after correction for multiple comparisons were found in FWC models, associating CST FA AH with grip force (*P* = 0.001) and pinch force (*P* < 0.001, Table 5, Fig. 2B). FWC leads to an increase of 19% in explained variance by CST FA AH in grip force and even 34% in pinch force compared to the model without FWC. FWC reduces RMSE of both models by 21 and 33%, respectively, clearly indicating that FWC significantly strengthens structure-behaviour relationships for ipsilesional CST microstructure as assessed by FA (Table 5). This is in line with LR-based model comparisons (grip force: LR-test *P* = 0.002, adj. *R*^2^_nested_ = 0.51, LR-test pinch force: *P*  < 0.001, adj. *R*^2^_nested_ = 0.69). For illustration, Fig. 2B plots *measured* vs. *predicted* grip forces and pinch forces for CST FA AH with and without FWC. Modelling results for all diffusion metrics including AD and RD values are given in Supplementary Table 4 (no significant results for AD and RD after full FDR correction).

**Table 5:** Association between tract-related microstructure of the corticospinal tract and behavioural scores

Outcome	Predictor	Model 1 | No FWC			Model 2 | FWC		
		Coef.	*P*	Adj. *R*²	Coef.	*P*	Adj. *R*²
Grip force AH	CST FA AH	68.30	**0.036^#^**	0.51	66.11	0.001*^r^	0.70
	CST FA UH	−46.08	0.343	0.37	55.83	0.150	0.42
	CST FW AH	–	–	–	−99.01	0.177	0.41
	CST FW UH	–	–	–	46.41	0.491	0.35
Pinch force AH	CST FA AH	12.49	0.154	0.38	17.62	<0.001*^r^	0.72
	CST FA UH	−13.59	0.272	0.34	16.11	0.165	0.38
	CST FW AH	–	–	–	−10.24	0.585	0.30
	CST FW UH	–	–	–	16.44	0.324	0.33
NHP AH	CST FA AH	0.88	0.091	0.33	0.44	0.278	0.24
	CST FA UH	−0.52	0.561	0.19	0.21	0.731	0.18
	CST FW AH	–	–	–	−2.30	**0.047^#^**	0.38
	CST FW UH	–	–	–	2.43	**0.015^#^**	0.47
UEFM	CST FA AH	40.32	0.185	0.18	36.39	0.080	0.25
	CST FA UH	−59.54	0.190	0.17	13.39	0.706	0.08
	CST FW AH	–	–	–	−50.24	0.481	0.10
	CST FW UH	–	–	–	142.08	**0.014^#^**	0.39

Coefficients (Coef.) are given incl. *P*-values (within regression model) for individual models for the 4 outcome variables (dependent variable) and CST FA of the affected hemisphere/hand (AH) or unaffected hemisphere/hand (UH). Middle column: Model 1 without FWC (i.e. only uncorrected FA values), right column: Model 2 with FWC applied (i.e. only corrected FA values). CST-related FW values were also used in separate models. Adj. *R*^2^ are given for the individual final models. For model specifications please see the statistics section. *Indicates significant predictors after FDR correction of all *P*-values for multiple testing for 24 tests. ^#^Indicates significant predictors based on uncorrected *P*-values in models which reached overall significance. Adj. *R*^2^ of baseline models (e.g. Grip force AH ∼ Grip force UH + age) were 0.37 for Grip force AH, 0.32 for Pinch force AH, 0.23 for NHP AH and 0.13 for UEFM (age as the only independent variable). ^r^FWC leads to a reduction of RMSE by 21% from 8.03 to 6.36 for grip force and 33% from 2.18 to 1.46 for pinch force. LR-based model comparisons reveal significant model improvement under FWC for both outcomes and CST FA AH.

Similar analyses in the healthy participants showed that already models without CST diffusion measures exhibited a very high degree of explained variance for grip force (adj. *R*^2^ = 0.91), pinch force (adj. *R*^2^ = 0.72) and NHP (adj. *R*^2^ = 0.76) due to the amount of collinearity between functional scores of both hands in healthy individuals. After correction for multiple testing, CST FA AH without FWC was still significantly positively related to grip force (*P* = 0.006), and CST FA AH without (*P* < 0.001) and with FWC (*P* = 0.008) was related to pinch force variability (data not shown). However, when omitting the functional state of the contralateral hand, these three models did not reach significance anymore. This argues that the results are very likely to be driven by the functional state of the other hand. Hence, these findings should be interpreted with caution. A further analysis of the relationship between CST microstructure and motor function in healthy elderly participants was not in the scope of the present work.

### Combining free water corrected CST microstructure with TMS to infer motor outcome

Finally, in order to evaluate the potential of FWC to infer the behavioural state from CST microstructure and TMS measures of cortical excitability in stroke patients, we constructed baseline models which comprised age, CST AH FA (with and without FWC), CST FW of AH and UH, MEPmax AH, SLOPEmax AH and s50 AH to explain motor outcome. Where applicable, the functional score of UH was considered as well. Final models were allowed to include only *one* structural and *one* TMS parameter to avoid overfitting. Table 6 gives an overview over the final models. The analyses showed that only after FWC, CST microstructure was found to significantly improve inferences of motor outcome. Specifically, corrected CST AH FA was found to exert a significant influence in addition to MEPmax AH to explain grip force variability. For pinch force, CST AH FA contributed in addition to SLOPEmax AH. Gain in explained variance (increase in adj. *R*^2^) was 9% for both models. Hereby, the influence of CST microstructure was numerically larger than the TMS parameters based on estimated coefficients on normalized predictors (Table 6). An interesting finding was that FW of the unaffected CST seemed to add to the models for UEFM and NHP. Notably, for all models relevant multi-collinearity of the independent predictors was excluded by means of VIF.

**Table 6:** Inference of behavioural scores from combined diffusion metrics and information of cortical excitability

Outcome	Predictor	Model 1 | No FWC			Model 2 | FWC		
		Coef. ^#^	*P*	Adj. *R*²	Coef.^#^	*P*	Adj. *R*²
Grip force AH	Grip force UH	8.43	<0.001	0.66	6.41	0.001	0.75
	CST FA AH	–	–	–	4.75	0.024	–
	MEPmax AH	5.88	0.003	–	3.13	0.100	–
	SLOPEmax AH	–	–	–	–	–	–
Pinch force AH	Pinch force UH	1.95	<0.001	0.71	1.43	0.001	0.80
	CST FA AH	–	–	–	1.15	0.013	–
	MEPmax AH	–	**-**	–	–	–	–
	SLOPEmax AH	1.62	<0,001	–	0.96	0.025	–
NHP AH	NHP UH	0.07	0.032	0.69	0.06	0.012	0.81
	CST FW UH	–	–	–	0.06	0.010	–
	SLOPEmax AH	0.12	<0.001	–	0.10	<0.001	–
	Age	−0.07	0.018	–	−0.08	0.002	–
UEFM	CST FW UH	–	–	0.64	4.55	0.001	0.77
	MEPmax AH	–	–	–	5.58	<0.001	–
	SLOPEmax AH	6.66	<0.001	–	–	–	–
	Age	−4.11	<0.008	–	−4.19	0.001	–

Predictors of winning multiple linear regression models are given to explain grip force, pinch force, NHP and UEFM scores. Final models stem from stepwise backward model simplification. Baseline models included the following factors as independent predictors: UH (unaffected hand) outcome (except UEFM), age, CST AH (affected hand) FA with or without FWC, CST FW of AH and UH only for models 2, and the following three TMS parameters: MEPmax AH, SLOPEmax AH, s50 AH. To prevent overfitting, only one TMS parameter and one DTI parameter (FA, CST FA-FWC or CST FW) was allowed for final models. Best model was selected based on Akaike Information Criterion. ^#^Normalized values were used for the independent variables to achieve comparability of the relative influences of the individual predictors. *P*-values are uncorrected.

## Discussion

The main finding of the present study was that elimination of FW in DTI-based characterization of CST microstructure in chronic stroke patients strengthens associations with TMS-related measures of CST excitability and clinical scores of motor output. More specifically, there was a robust association between FWC FA of the ipsilesional CST and the slope of the TMS derived cortical excitability measure RC. The FWC FA explained additional 20% in RC variability compared with the non-FWC FA. Herewith the present data provided further evidence supporting the view that CST excitability depends on CST microstructure, a matter of debate in numerous previous reports. For clinical scores, FWC also led to the detection of significant correlations between ipsilesional CST FA and residual grip and pinch force variability which were not detectable without FWC in the present cohort. Finally, FWC also significantly improved multimodal regression models to infer motor outcome.

### Free water correction strengthens the link between CST microstructure and CST excitability after stroke

A number of studies in stroke patients have aimed at correlating microstructural properties of the damaged CST to electrophysiological measures of altered cortical excitability. For instance, Lotze et al.^6^ attempted to correlate TMS measures with the number of corticospinal streamlines passing through the posterior limb of the internal capsule (PLIC) and its proportional mean FA, but could not detect any correlations in 14 chronic stroke patients.^6^ Subsequently, Buetefisch et al.^5^ have similarly reported absent correlations between FA of the entire CST and various TMS parameters of excitability in 17 patients. They have discussed that the investigation of isolated M1 fibres would be needed to potentially uncover the hypothesized structure-function associations for the CST.^5^ In fact, already two years earlier, Potter-Baker et al.^7^ had used DTI to reconstruct CST originating from multiple cortical brain regions. Interestingly, they had found that the amount of asymmetry in CST FA of fibres emerging from premotor regions but not from M1 was related to selected RC properties. Notably, caution is advised given the small sample size of only 8 patients.^7^

Our data now indicate that FWC is helpful in inferring electrophysiological characteristics of ipsilesional corticospinal excitability from microstructural properties of the underlying structural pathways. In fact, FA estimation was conducted at the level of CP, remote from the lesion. Thus, oedema in or around the stroke lesion is unlikely to cause the contamination with FW, eliminated by FWC in our analyses. We rather argue that partial volume effects from CSF around the CP and the brain stem seems to be more likely to drive the present findings. Importantly, despite some uncorrected statistical signals for selected models (see Results section) we are convinced that whatever causes FW contamination is not determining function. Explained variance in our FWC-models ranged from 21% for maximum MEP to 54% for maximum RC slope. This indicates that a relevant amount of variability still remains unexplained and is likely to locate beyond CST microstructure investigated in the present report. For instance, unexplained variance might be attributed to stroke-related alterations in spinal or intra-cortical functioning of neuronal assemblies.^18^ For instance, excitability of lower motor neurons, assessed by means of F-wave properties in nerve conduction studies, such as persistence or response amplitudes, were found to be reduced after stroke.^19^ Hence, inter-subject variability in these measures might further help to improve the present models.

Unexplained variance might also be attributed to the fact that, as discussed by previous reports, only M1- or even non-M1 derived CST subcomponents might specifically influence TMS measures. Our CST templates are derived from DTI seeding in M1 with a clear relation to hand function.^16^ However, secondary CST contributions were not investigated in the present study. Thus, we cannot contribute to the discussion in Potter-Baker et al.^7^ at this stage. Notably, CST subcomponents can be separately assessed at PLIC level and above,^7^^,^^20^ but with the disadvantage of direct lesion effects which complicate the interpretation of diffusion-based properties of CST microstructure. Studies in monkeys and humans have indicated that 40–60% of CST fibres originate from M1 and only 15–25% from supplementary motor areas.^21^ Therefore, we argue that the majority of information is likely to be contributed by CST emerging from M1 as our data show that already *overall* CST, assessed at CP, significantly relates to TMS excitability. We are not convinced that only microstructure of non-M1 derived fibres should primarily shape RC properties in chronic stroke patients but admit that more advanced high-resolution DTI will be needed to disentangle primary and secondary CST more precisely and relate their microstructure to measures of excitability to finally solve this open question. Interestingly, recent diffusion spectrum imaging has provided novel insights into the topography of different CST fibres even at CP level.^22^

For healthy participants the analysis did not show any significant associations between CST microstructure—neither with nor without FWC—and TMS parameters. Overall, this is in good agreement with previous reports.^23^^,^^24^ Hence, CST microstructure appears not to be significantly contributing to inter-subject variability in measures of cortical excitability in healthy participants. Spinal properties and differences in intra-cortical functioning or neurotransmission^25^ might be more relevant influential factors.

### Free water correction strengthens the association between CST microstructure and motor outcome after stroke

In terms of clinical scores and motor behaviour we found that FWC significantly strengthens the positive association between mean FA of the ipsilesional CST and residual motor output, taking the unaffected hand into account. Roughly, FWC resulted in additional 19–34% explanation of inter-subject variances. The potential of the present approach is also illustrated when comparing the actual levels of explained variances of 70% for grip force and 72% for pinch force after FWC with recent analyses showing correlation coefficients of moderate 0.38–0.47 (estimated *R*^2^ = 14–22%) for asymmetry indices of FW corrected FA of the CST at the level of the CP.^10^

Many previous studies have used proportional functional scores, relating the affected hand to the unaffected hand. However, there is relevant evidence that also the unaffected hands might undergo functional changes after stroke.^26^^,^^27^ Therefore, our models included the motor function of the unaffected hand as *independent* covariates. *Posthoc*, we evaluated how the models for CST FA AH would perform in reduced models with *proportional* grip force and pinch force values (affected hand/unaffected hand) as the dependent variables. Grip ratio could be still significantly explained by CST FA AH without FWC (adj. *R*^2^ = 0.30, *P* = 0.02) with higher FA values associated with higher grip force values (*P* = 0.002). In contrast, without FWC, pinch ratio values could not be reliably explained by the CST microstructure (adj. *R*^2^ = 0.06, n.s.). In line with our main findings, FWC resulted in improved grip and pinch force explanation (grip ratio: Adj. *R*^2^ = 0.41, *P* = 0.007; pinch ratio: Adj. *R*^2^ = 0.52, *P* = 0.002) indicating that our modelling results are not driven by overfitting and inclusion of the unaffected hand as a covariate.

Notably, similar findings can be also found for electrophysiology and SLOPEmax when reducing the TMS models to proportional SLOPEratio values for RC description. Importantly though, excitability changes have been reported repeatedly not only for the ipsilesional but also the contralesional hemispheres which is likely to complicate the interpretation of RC ratio values.^28^ Nevertheless, also these *posthoc* analyses evidenced that CST FA AH still related to SLOPEratio already without FWC (adj. *R*^2^ = 0.31, *P* = 0.02), but significantly better with FWC (adj. *R*^2^ = 0.54, *P* = 0.001). FWC leads to an increase of 23% in explained variance.

### Free water correction strengthens multimodal models to infer motor outcome after stroke

CST microstructure and TMS parameter were finally combined to explain variability in motor outcome after stroke. We found that only after FWC, model optimization resulted in the inclusion of CST microstructure as a relevant predictor to infer residual motor outcome in our cohort. Gain in explained variance was around 9%. Moreover, these analyses revealed that multi-collinearity did not limit the potential of FWC-derived microstructure in multimodal models. Comparable modelling to explain variance in motor output after stroke has been successfully conducted for CST lesion load and interhemispheric connectivity^29^ or even both parameters and cortical excitability.^4^

### Free water correction influences group comparisons regarding CST microstructure

We also performed group comparisons of CST microstructure with and without FWC and found significantly lower FA, independently from FWC, in the CST of the ipsilesional hemisphere, in line with the broad body of evidence in the literature.^1^ However, when contrasting FWC with groups only (GROUP*FWC interaction), we found that CST FA across hemispheres was also lower in stroke patients. This would argue that CST of *both* hemispheres undergo deterioration of white matter microstructure, a finding which was only detected in FW corrected CST FA and not in uncorrected FA values. This supports previous reports showing that white matter alterations do not only affect the ipsilesional but also contralesional hemisphere and CST (e.g. Schaechter et al.^30^). The FA decrease in ipsilesional FA was paralleled by an increase in FW volume in stroke patients. Lastly, we also found a disease-independent effect of age with increases in tract-related FW with increasing age, already known from recent reports in large cohorts of healthy participants.^31^

### Limitations

There are several limitations worth to note. First, we have used CST templates to assess its microstructure at the level of the upper CP. This approach has been repeatedly used^13^^,^^16^^,^^17^^,^^32^ and is in line with suggestions in the literature.^33^^,^^34^ The individual CST trajectories might vary in the patients which could influence the present results. Other regions of interest, such as PLIC, or even the analysis of the whole extent of the CST with its lesioned parts might alter the present findings.^7^^,^^10^ A slice-by-slice analysis would further increase the risk of false positives. In fact, the actual statistical modelling was geared to achieve high specificity at the cost of limited sensitivity by applying consequent correction for multiple comparisons. Second, this study included patients in the chronic stage of recovery. It remains an open question whether our findings will also hold true for patients earlier after stroke. Also, only patients with supratentorial stroke lesions have been included. Hence, whether patients, for example with pontine lesions would show similar structure-function associations would be a topic for upcoming studies. Finally, estimating the FW model from multi-shell data would be more accurate than the single-shell data which we used in the present study.^12^ The present findings should be tested based on more elaborated diffusion MRI sequences.

#### Summary and Outlook

FW elimination has been increasingly investigated in DTI and various fields of clinical neuroscience including research in small vessel disease,^35^ healthy aging,^31^ dementia^36^ or movement disorders.^37^ Technically, it has been shown that this approach significantly improves test-retest reproducibility.^38^ For stroke research, the present report contributes to the hitherto extensively discussed relationship between CST structure and electrophysiology in stroke patients. Together with the secondary findings of our previous report,^13^ our dataset shows that CST microstructure influences corticospinal excitability. In fact, while this relationship seems to be intuitive, its evidence however was still missing. Together with the clinical associations our data also illustrate that FWC can help DTI studies to come closer to the ground truth of neuroplasticity and structural network alterations after stroke. With regard to stroke-related changes of cortical excitability the present results significantly reduce the amount of unexplained variance. Using multimodal approaches including spectroscopy,^25^ TMS^39^ or morphometric analyses^40^ future studies might aim to further explore this residual variance to better understand function and malfunction of the corticospinal motor network after stroke.

## Supplementary material

Supplementary material is available at *Brain Communications* online.

## Funding

Werner Otto Stiftung (4/90 to R.S.), National Institutes of Health (NIH P41EB015902 to O.P.) and the German Research Foundation (SFB 936-C1 to C.G.).

## Competing interests

The authors report no competing interests.

## Supplementary Material

fcab034_Supplementary_Data

## Abbreviations

AD =: axial diffusivity
CST =: corticospinal tract
DTI =: diffusion-tensor-imaging
FA =: fractional anisotropy
FW =: free water
FWC =: free water correction
M1 =: primary motor cortex
MEP =: motor-evoked potential
MSO =: maximum stimulator output
RC =: recruitment curve
RD =: radial diffusivity
RMT =: resting motor threshold
TMS =: transcranial magnetic stimulation
UEFM =: Fugl-Meyer assessment of the upper extremity

## References

[1] Koch P, Schulz R, Hummel FC. Structural connectivity analyses in motor recovery research after stroke. Ann Clin Transl Neurol. 2016;3:233–244. 27042683 10.1002/acn3.278 PMC4774263

[2] Feng W, Wang J, Chhatbar PY, et al Corticospinal tract lesion load: An imaging biomarker for stroke motor outcomes. Ann Neurol. 2015;78:860–870. 26289123 10.1002/ana.24510 PMC4715758

[3] Jin J-F, Guo Z-T, Zhang Y-P, Chen Y-Y. Prediction of motor recovery after ischemic stroke using diffusion tensor imaging: A meta-analysis. World J Emerg Med. 2017;8:99–105. 28458752 10.5847/wjem.j.1920-8642.2017.02.003 PMC5409242

[4] Volz LJ, Sarfeld A-S, Diekhoff S, et al Motor cortex excitability and connectivity in chronic stroke: A multimodal model of functional reorganization. Brain Struct Funct. 2015;220:1093–1107. 24415059 10.1007/s00429-013-0702-8

[5] Buetefisch CM, Pirog RK, Haut MW, et al Abnormally reduced primary motor cortex output is related to impaired hand function in chronic stroke. J Neurophysiol. 2018;120:1680–1694. [10.1152/jn.00715.2017] 29924707 10.1152/jn.00715.2017 PMC6230804

[6] Lotze M, Beutling W, Loibl M, et al Contralesional motor cortex activation depends on ipsilesional corticospinal tract integrity in well-recovered subcortical stroke patients. Neurorehabil Neural Repair. 2012;26:594–603. 22140195 10.1177/1545968311427706

[7] Potter-Baker KA, Varnerin NM, Cunningham DA, et al Influence of corticospinal tracts from higher order motor cortices on recruitment curve properties in stroke. Front Neurosci. 2016;10:79. 10.3389/fnins.2016.00079 PMC4781847 27013942

[8] Stinear CM, Barber PA, Smale PR, Coxon JP, Fleming MK, Byblow WD. Functional potential in chronic stroke patients depends on corticospinal tract integrity. Brain. 2007;130:170–180. 17148468 10.1093/brain/awl333

[9] Pasternak O, Sochen N, Gur Y, Intrator N, Assaf Y. Free water elimination and mapping from diffusion MRI. Magn Reson Med. 2009;62:717–730. 19623619 10.1002/mrm.22055

[10] Archer DB, Patten C, Coombes SA. Free-water and free-water corrected fractional anisotropy in primary and premotor corticospinal tracts in chronic stroke. Hum Brain Mapp. 2017;38:4546–4562. 28590584 10.1002/hbm.23681 PMC6866851

[11] Metzler-Baddeley C, O’Sullivan MJ, Bells S, Pasternak O, Jones DK. How and how not to correct for CSF-contamination in diffusion MRI. Neuroimage. 2012;59:1394–1403. 21924365 10.1016/j.neuroimage.2011.08.043

[12] Filatova OG, van Vliet LJ, Schouten AC, Kwakkel G, van der Helm FCT, Vos FM. Comparison of multi-tensor diffusion models’ performance for white matter integrity estimation in chronic stroke. Front Neurosci. 2018;12:247. 29740269 10.3389/fnins.2018.00247 PMC5925961

[13] Guder S, Frey BM, Backhaus W, et al The influence of cortico-cerebellar structural connectivity on cortical excitability in chronic stroke. Cereb Cortex. 2020;30:1330–1344. 31647536 10.1093/cercor/bhz169

[14] Devanne H, Lavoie BA, Capaday C. Input-output properties and gain changes in the human corticospinal pathway. Exp Brain Res. 1997;114:329–338. 9166922 10.1007/pl00005641

[15] Behrens TEJ, Woolrich MW, Jenkinson M, et al Characterization and propagation of uncertainty in diffusion-weighted MR imaging. Magn Reson Med. 2003;50:1077–1088. 14587019 10.1002/mrm.10609

[16] Schulz R, Koch P, Zimerman M, et al Parietofrontal motor pathways and their association with motor function after stroke. Brain. 2015;138:1949–1960. 25935722 10.1093/brain/awv100

[17] Schulz R, Frey BM, Koch P, et al Cortico-cerebellar structural connectivity is related to residual motor output in chronic stroke. Cereb Cortex. 2017;27:635–645. 26508336 10.1093/cercor/bhv251

[18] McDonnell MN, Stinear CM. TMS measures of motor cortex function after stroke: A meta-analysis. Brain Stimul. 2017;10:721–734. 28385535 10.1016/j.brs.2017.03.008

[19] Naseri M, Petramfar P, Ashraf A. Effect of motor imagery on the F-wave parameters in hemiparetic stroke survivors. Ann Rehabil Med. 2015;39:401. (401):26161346 10.5535/arm.2015.39.3.401 PMC4496511

[20] Schulz R, Park C-H, Boudrias M-H, Gerloff C, Hummel FC, Ward NS. Assessing the integrity of corticospinal pathways from primary and secondary cortical motor areas after stroke. Stroke. 2012;43:2248–2251. 22764214 10.1161/STROKEAHA.112.662619 PMC3477824

[21] Welniarz Q, Dusart I, Roze E. The corticospinal tract: Evolution, development, and human disorders. Dev Neurobiol. 2017;77:810–829. 27706924 10.1002/dneu.22455

[22] Liu J, Wang C, Qin W, et al Corticospinal fibers with different origins impact motor outcome and brain after subcortical stroke. Stroke. 2020;51:2170–2178. 32568657 10.1161/STROKEAHA.120.029508

[23] Klöppel S, Bäumer T, Kroeger J, et al The cortical motor threshold reflects microstructural properties of cerebral white matter. Neuroimage. 2008;40:1782–1791. 18342540 10.1016/j.neuroimage.2008.01.019

[24] Hübers A, Klein JC, Kang J-S, Hilker R, Ziemann U. The relationship between TMS measures of functional properties and DTI measures of microstructure of the corticospinal tract. Brain Stimul. 2012;5:297–304. 22037122 10.1016/j.brs.2011.03.008

[25] Stagg CJ, Bestmann S, Constantinescu AO, et al Relationship between physiological measures of excitability and levels of glutamate and GABA in the human motor cortex. J Physiol. 2011;589:5845–5855. 22005678 10.1113/jphysiol.2011.216978 PMC3249054

[26] Balcı NC, Dogru E, Aytar A, Gokmen O, Depreli O. Comparison of upper extremity function, pain, and tactile sense between the uneffected side of hemiparetic patients and healthy subjects. J Phys Ther Sci. 2016;28:1998–2001. 27512250 10.1589/jpts.28.1998 PMC4968492

[27] Yu Z, Zhang L, Li P, Mao Z, Qi X, Zou J. Changes in motor function in the unaffected hand of stroke patients should not be ignored. Neural Regen Res. 2014;9:1323–1328. 25221586 10.4103/1673-5374.137581 PMC4160860

[28] Beaulieu L-D, Milot M-H. Changes in transcranial magnetic stimulation outcome measures in response to upper-limb physical training in stroke: A systematic review of randomized controlled trials. Ann Phys Rehabil Med. 2018;61:224–234. 28579362 10.1016/j.rehab.2017.04.003

[29] Lam TK, Binns MA, Honjo K, et al Variability in stroke motor outcome is explained by structural and functional integrity of the motor system. Sci Rep. 2018;8:9480- 29930399 10.1038/s41598-018-27541-8 PMC6013462

[30] Schaechter JD, Fricker ZP, Perdue KL, et al Microstructural status of ipsilesional and contralesional corticospinal tract correlates with motor skill in chronic stroke patients. Hum Brain Mapp. 2009;30:3461–3474. 19370766 10.1002/hbm.20770 PMC2780023

[31] Chad JA, Pasternak O, Salat DH, Chen JJ. Re-examining age-related differences in white matter microstructure with free-water corrected diffusion tensor imaging. Neurobiol Aging. 2018;71:161–170. 30145396 10.1016/j.neurobiolaging.2018.07.018 PMC6179151

[32] Rimmele DL, Frey BM, Cheng B, et al Association of extrapyramidal tracts’ integrity with performance in fine motor skills after stroke. Stroke. 2018;49:2928–2932. 30571408 10.1161/STROKEAHA.118.022706

[33] Park C, Kou N, Boudrias M-H, Playford ED, Ward NS. Assessing a standardised approach to measuring corticospinal integrity after stroke with DTI. NeuroImage Clin. 2013;2:521–533. 24179804 10.1016/j.nicl.2013.04.002 PMC3777681

[34] Hirai KK, Groisser BN, Copen WA, Singhal AB, Schaechter JD. Comparing prognostic strength of acute corticospinal tract injury measured by a new diffusion tensor imaging based template approach versus common approaches. J Neurosci Methods. 2016;257:204–213. 26386285 10.1016/j.jneumeth.2015.09.005 PMC4666681

[35] Duering M, Finsterwalder S, Baykara E, et al Free water determines diffusion alterations and clinical status in cerebral small vessel disease. Alzheimer’s Dement. 2018;14:764–774. 29406155 10.1016/j.jalz.2017.12.007 PMC5994358

[36] Dumont M, Roy M, Jodoin P-M, et al; Alzheimer's Disease Neuroimaging Initiative. Free Water in White Matter Differentiates MCI and AD From Control Subjects. Front Aging Neurosci. 2019;11:10.3389/fnagi.2019.00270 PMC6783505 31632265

[37] Ofori E, Pasternak O, Planetta PJ, et al Longitudinal changes in free-water within the substantia nigra of Parkinson’s disease. Brain. 2015;138:2322–2331. 25981960 10.1093/brain/awv136 PMC4840947

[38] Albi A, Pasternak O, Minati L, et al PharmaCog Consortium. Free water elimination improves test–retest reproducibility of diffusion tensor imaging indices in the brain: A longitudinal multisite study of healthy elderly subjects. Hum Brain Mapp. 2017;38:12–26. 27519630 10.1002/hbm.23350 PMC5493991

[39] Auriat AM, Neva JL, Peters S, Ferris JK, Boyd LA. A review of transcranial magnetic stimulation and multimodal neuroimaging to characterize post-stroke neuroplasticity. Front Neurol. 2015;6:226. 26579069 10.3389/fneur.2015.00226 PMC4625082

[40] Cheng B, Dietzmann P, Schulz R, et al Cortical atrophy and transcallosal diaschisis following isolated subcortical stroke. J Cereb Blood Flow Metab. 2020;40:611–621. 30782059 10.1177/0271678X19831583 PMC7026841

