# Supplementary material for: Strengthened structure–function relationships of the corticospinal tract by free water correction after stroke
Source: Brain Commun. 2021 Apr 28;3(2):fcab034. doi: 10.1093/braincomms/fcab034 (PMC8088790; doi:10.1093/braincomms/fcab034)
Supplement: fcab034_Supplementary_Data [file fcab034_supplementary_data.pdf]

Supplementary Material

Strengthened structure-function relationships of the corticospinal tract by free water correction after stroke

Stephanie Guder[1], Ofer Pasternak[2], Christian Gerloff[1], Robert Schulz[1]

[1] Department of Neurology, University Medical Centre Hamburg-Eppendorf, Hamburg, Germany

[2] Departments of Psychiatry and Radiology, Brigham and Women's Hospital, Harvard Medical School, Boston, MA, USA

Methods

TMS data acquisition and analysis

TMS data acquisition was conducted using a Magstim 200 magnetic stimulator with a figure of eight coil with a 70 mm wing diameter and EMG electrodes placed over the first dorsal interosseous muscle (FDI) on both hands in a belly-tendon montage. Methods are described in detail in our previous report (Guder *et al.*, 2020). In brief, at the MEP hot spot over M1, the resting motor threshold (RMT, minimum stimulus intensity to evoke an MEP of > 0.05 mV in ≥ 5/10 trials), was determined to the nearest 1% of the maximum stimulator output (MSO). To obtain properties of the MEP recruitment curves (RC, Devanne *et al.*, 1997) we used blocks of 11 stimuli with an inter-stimulus-interval of 8 seconds and an inter-block break of 40 seconds for each intensity value, ranging from 90% to 160% of RMT. The first trial in each block was discarded. The order of the stimuli intensities was pseudo-randomized in order to avoid hysteresis effects (Möller *et al.*, 2009). RCs were measured at both hemispheres. The information, whether the affected hemisphere was measured first or second, was pseudorandomized before measurement to control for fatigue in the later analysis. In the control group we alternated the beginning between the left and right hemisphere. Data analysis was conducted using Signal software 4.05 (Cambridge Electronic Design, Cambridge, UK). Trials with increased EMG background activity were excluded from the final analysis following previous reports (Buetefisch *et al.*, 2018). Fitting of RC was based on at least 5 trials per stimulus intensity from 90-160% RMT except for one patient and one control participants in which 160% RMT could not be reached due to high RMT and increased stimulation intensities at 160% (>90% of total MSO). RC data are plotted vs. MEP size, noted as peak-to-peak amplitude. R Studio was used to fit the three parameter sigmoid Boltzman function to each individual RC, thereby estimating MEPmax (plateau of RC), SLOPEmax (slope of RC, straight line fitted to RC at its inflection point) and stimulus intensity s50 to obtain a response 50% of the maximum. Further details are given in the original report (Guder *et al.*, 2020).

Brain Imaging

Available imaging data included high-resolution T1-weighted anatomical and diffusion weighted images, acquired on a 3-T Siemens Skyra MRI scanner (Siemens, Erlangen, Germany) and a 32-channel head coil. Diffusion-weighted images consisted of 75 axial slices covering the whole brain with gradients (b=1500 s/mm2) applied along 64 non-collinear

directions with the following sequence parameters: repetition time (TR)=10.000 ms, echo time (TE)=82 ms, field of view (FOV)=256 mm, slice thickness (ST)=2 mm, in-plane resolution (IPR)=2 × 2 mm. T1-weighted images were acquired using a three-dimensional magnetization-prepared, rapid acquisition gradient-echo sequence (MPRAGE) with the following parameters: TR=2500 ms, TE=2.12 ms, FOV=240 mm, 256 coronal slices with a voxel size of 0.8 x 0.8 x 0.9 mm. Brain imaging was conducted using the FSL software package 5.0.2.2 (http://www.fmrib.ox.ac.uk/fsl). Brain extraction was performed after correcting for eddy currents and head motion. The resulting 4D volumes was then used as inputs in two different procedures: (1) DTIFIT, implemented in FSL, was used to calculate fractional anisotropy (FA) maps by fitting the diffusion tensor model at each voxel (Behrens *et al.*, 2003). Based on the tensor information, maps for alternative diffusion metrics that are mean axial (AD) and radial diffusivity (RD) were also calculated. (2) A custom written MATLAB script (ran on R2016a, The Mathworks, Natick, MA, USA) (Pasternak *et al.*, 2009) was used to estimate free water (FW) maps and FA maps, AD and RD maps after FWC. In brief, a bi-tensor model is calculated to predict signal attenuation in the presence of FW contamination. The model includes two different compartments: The first compartment estimates the fractional volume of FW, which is modelled as an isotropic tensor with a fixed diffusivity. The other compartment uses a diffusion-tensor to model water molecules in the vicinity of tissue membranes, from which FWC DTI measures are calculated (Pasternak *et al.*, 2009). The uncorrected individual FA-maps were subsequently registered non-linearly by means of FSL *flirt* and *fnirt* to the Montreal Neurological Institute (MNI) standard space. The resulting transformation was applied to all other diffusion metrics with and without FWC. To quantify CST microstructure, we used available binarized tract templates to read out tract-related mean FA, AD and RD values at the level of the mesencephalon to the cerebral peduncles (z=-25 to -20). These templates were available after performing individual tract reconstructions in 26 healthy older participants by means of probabilistic tractography with subsequent thresholding the output distribution (by 0.01% - 2% of successful streamlines) and binarizing them according to an overlap of at least 65% of the group (Schulz *et al.*, 2015, 2017).

Controls demographic and clinical characteristics

	Gender	Age	DoHe	AfHe	NHP		Gripforce		Pinchforce	
					AH	UH	AH	UH	AH	UH
1	M	79	L	L	0.7	0.7	38.7	39.3	1.0	8.5
2	F	78	L	L	0.5	0.5	6.7	9.0	0.7	4.3
3	M	77	L	L	0.6	0.6	46.0	44.0	1.0	9.5
4	F	73	L	R	0.6	0.7	18.7	20.0	0.9	4.7
5	M	59	L	R	0.8	0.8	40.7	45.7	0.9	9.2
6	M	57	L	L	0.7	0.8	38.7	36.0	1.1	9.5
7	M	75	L	R	0.6	0.8	37.0	42.3	0.9	6.5
8	M	65	L	L	1.1	0.8	45.0	39.7	1.1	10.0
9	M	71	L	L	0.7	0.7	31.0	29.3	1.1	3.2
10	M	68	L	L	0.7	0.7	30.3	26.3	1.2	5.8
11	M	69	L	L	0.7	0.7	44.3	38.0	1.2	7.3
12	M	57	R	R	1.1	1.0	47.0	40.7	1.2	10.3
13	M	58	L	L	1.0	0.9	44.0	36.7	1.2	8.3
14	M	55	L	L	0.9	0.8	38.0	32.0	1.2	3.3
15	M	69	L	R	0.6	0.6	29.7	29.3	1.0	9.2
16	M	60	L	L	1.0	0.8	44.3	43.0	1.0	9.3
17	F	65	L	L	0.6	0.5	11.7	13.3	0.9	5.7
Mean±SEM	M:15	66.8±2.0	L:16	L:12	0.8±0.04	0.7±0.03	34.8±3.0	33.2±2.6	7.3±0.6	7.2±0.6

SOM Table 1 | Clinical data controls

Gender (m=male; f=female) and age (in years), dominant (DoHe) and affected (AfHe) hemisphere (L=left; R=right), NHP=Nine-hole-peg performance (in pegs per seconds; AH=affected hand; UH=unaffected hand). Absolute pinch and grip force values (both in kg). Group average is given in mean ± standard error of the mean (SEM).

Results

Free water correction and CST microstructure

Results for CST FA are described in the main text. For CST AD, there was only a significant main effect for FWC indicating higher AD values after FWC across groups and hemispheres ($p<0.0001$). For CST RD, there was a significant GROUP*SIDE interaction ($F_{1,105}=9.7$, $p=0.002$). Independent from FWC, CST RD values were significantly higher in AH (est. mean 0.00040) compared to UH (est. mean 0.00037, p=0.003) in stroke patients.

	Tract	Stroke patients			Healthy controls		
		Est. mean	95% CI		Est. mean	95% CI	
			Lower	Upper		Lower	Upper
No FWC	FA AH	0,57	0,53	0,60	0,63	0,59	0,67
	FA UH	0,61	0,57	0,65	0,62	0,58	0,66
	AD AH	0,00129	0,00125	0,00134	0,00130	0,00125	0,00134
	AD UH	0,00130	0,00125	0,00134	0,00129	0,00124	0,00134
	RD AH	0,00051	0,00048	0,00054	0,00044	0,00041	0,00047
	RD UH	0,00047	0,00044	0,00050	0,00046	0,00042	0,00049
FWC	FA AH	0,55	0,51	0,59	0,72	0,68	0,76
	FA UH	0,62	0,58	0,66	0,71	0,67	0,75
	AD AH	0,00108	0,00104	0,00113	0,00113	0,00108	0,00118
	AD UH	0,00112	0,00107	0,00116	0,00111	0,00107	0,00116
	RD AH	0,00029	0,00026	0,00032	0,00027	0,00024	0,00030
	RD UH	0,00027	0,00024	0,00030	0,00027	0,00024	0,00030
FW	FW AH	0,25	0,23	0,27	0,20	0,18	0,22
	FW UH	0,21	0,19	0,23	0,22	0,20	0,24

SOM Table 2 | CST microstructural properties for AD and RD

Estimated means with 95% confidence intervals (CI) are given for diffusion properties of the CST of the affected (AH) and unaffected (UH) hemispheres. FWC free water correction, FW free water volume. For group comparisons, please refer to the text. As triple interactions were not significant, comparisons on individual values were not conducted.

Free water correction for CST microstructure and cortical excitability

		Model 1 | No FWC			Model 2 | FWC		
Outcome	Tract	Coef.	*P*	Adj. R^2	Coef.	*P*	Adj. R^2
MEPmax AH	FA AH	2,25	0,108	0,11	1,96	0,039	0,21
	FA UH	-1,25	0,583	-0,06	2,23	0,197	0,04
	AD AH	1274,79	0,385	-0,02	1525,96	0,269	0,01
	AD UH	104,57	0,942	-0,08	-379,65	0,827	-0,08
	RD AH	-2143,20	0,162	0,06	-3153,45	0,212	0,04
	RD UH	767,32	0,676	-0,07	2998,33	0,434	-0,03
	FW AH				-4,07	0,213	0,04
	FW UH				0,13	0,967	-0,08
SLOPEmax AH	FA AH	3,37	**0,017**[#]	0,34	2,97	**0,001**[*r]	0,54
	FA UH	-1,51	0,524	0,02	3,00	0,098	0,18
	AD AH	2276,83	0,126	0,15	2745,77	0,048	0,25
	AD UH	346,36	0,815	0,00	-259,25	0,882	0,00
	RD AH	-2994,08	0,056	0,23	-5375,72	0,043	0,26
	RD UH	1410,08	0,479	0,03	3251,36	0,410	0,04
	FW AH				-4,53	0,176	0,12
	FW UH				1,78	0,597	0,02
s50 AH	FA AH	-0,10	0,711	0,33	-0,05	0,800	0,33
	FA UH	0,30	0,459	0,35	0,25	0,406	0,36
	AD AH	187,11	0,506	0,35	28,34	0,919	0,33
	AD UH	-244,73	0,361	0,37	1,60	0,996	0,33
	RD AH	243,48	0,406	0,36	29,67	0,951	0,33
	RD UH	-505,62	0,118	0,44	-365,12	0,587	0,34
	FW AH				0,84	0,161	0,42
	FW UH				-1,20	**0,018**[#]	0,55

SOM Table 3 | Tract-related microstructure of the CST and TMS measures

Coefficients are given incl. *P*-values of tract of interest (within regression model) in individual models for the 3 outcome variables (dependent variable) and CST of the affected hemisphere (AH) or unaffected hemisphere (UH) and for the 3 different DTI measures, that are tract-related FA, AD and RD. Middle column: model 1 without FWC, right column: model 2 with FWC applied. CST-related FW values were also used in separate models. Adj. R^2 are given for the complete final models. For model specifications please see the statistics section. * indicates significant predictors after FDR correction of all *P*-values for multiple testing for 42 test. [#] indicates significant predictors based on uncorrected *P*-values in models which reached overall significance. Adj. R^2 of baseline models (e.g. MEPmax AH ~ MEPmax UH + age) were -0.01 for MEPmax AH, 0.06 for SLOPEmax AH and 0.37 for s50 AH. [r] FWC leads to a reduction of RMSE by 17% from 0.35 to 0.29. LR-based model comparison reveals a significant model improvement under FWC.

Free water correction for CST microstructure and behaviour

Outcome	Tract	Model 1 | No FWC Coef.	*P*	Adj. R^2	Model 2 | FWC Coef.	*P*	Adj. R^2
Grip force AH	FA AH	68,30	**0,036**[#]	0,51	66,11	**0,001***[r]	0,70
	FA UH	-46,08	0,343	0,37	55,83	0,150	0,42
	AD AH	53005,26	0,110	0,44	50661,85	0,108	0,44
	AD UH	24845,87	0,433	0,36	-3694,45	0,925	0,33
	RD AH	-57031,06	0,103	0,45	-117546,55	**0,048**[#]	0,50
	RD UH	47854,91	0,228	0,40	73921,14	0,364	0,37
	FW AH				-99,01	0,177	0,41
	FW UH				46,41	0,491	0,35
Pinch force AH	FA AH	12,49	0,154	0,38	17,62	**<0,001***[r]	0,72
	FA UH	-13,59	0,272	0,34	16,11	0,165	0,38
	AD AH	17562,23	**0,040**[#]	0,47	12069,37	0,160	0,38
	AD UH	7428,17	0,349	0,33	2121,03	0,833	0,28
	RD AH	-6474,90	0,479	0,31	-21919,88	0,173	0,37
	RD UH	13094,28	0,188	0,37	24660,46	0,235	0,35
	FW AH				-10,24	0,585	0,30
	FW UH				16,44	0,324	0,33
NHP AH	FA AH	0,88	0,091	0,33	0,44	0,278	0,24
	FA UH	-0,52	0,561	0,19	0,21	0,731	0,18
	AD AH	175,63	0,749	0,18	632,43	0,213	0,26
	AD UH	979,47	0,058	0,37	467,25	0,495	0,20
	RD AH	-1042,49	0,060	0,36	-911,63	0,336	0,23
	RD UH	1040,28	0,119	0,31	563,61	0,706	0,18
	FW AH				-2,30	**0,047**[#]	0,38
	FW UH				2,43	**0,015**[#]	0,47
UEFM	FA AH	40,32	0,185	0,18	36,39	0,080	0,25
	FA UH	-59,54	0,190	0,17	13,39	0,706	0,08
	AD AH	36344,53	0,242	0,15	37568,85	0,200	0,17
	AD UH	30635,88	0,303	0,14	-1840,62	0,959	0,07
	RD AH	-32848,20	0,324	0,13	-52970,46	0,328	0,13
	RD UH	70990,56	**0,049**[#]	0,29	79123,69	0,303	0,14
	FW AH				-50,24	0,481	0,10
	FW UH				142,08	**0,014**[#]	0,39

SOM Table 4 | Tract-related microstructure of the CST and behavioural scores

Coefficients are given incl. *P*-values (within regression model) for individual models for the 4 outcome variables (dependent variable) and CST of the affected hemisphere/hand (AH) or unaffected hemisphere/hand (UH) and for the 3 different DTI measures, that are tract-related FA, AD and RD. Middle column: model 1 without FWC, right column: model 2 with FWC applied. CST-related FW values were also used in separate models. Adj. R^2 are given for the individual final models. For model specifications please see the statistics section. * indicates significant predictors after FDR correction of all P-values for multiple testing for 56 test. [#] indicates significant predictors based on uncorrected P-values in models which reached overall significance. Adj. R^2 of baseline models (e.g. Grip force AH ~ Grip force UH + age) were 0.37 for Grip force AH, 0.32 for Pinch force AH, 0.23 for NHP AH and 0.13 for UEFM (age as the only independent variable). [r] FWC leads to a reduction of RMSE by 21% from 8.03 to 6.36 for grip force and 33% from 2.18 to 1.46 for pinch force. LR-based model comparisons reveal significant model improvement under FWC for both outcomes and CST FA AH.

References

Behrens TEJ, Woolrich MW, Jenkinson M, Johansen-Berg H, Nunes RG, Clare S, et al. Characterization and Propagation of Uncertainty in Diffusion-Weighted MR Imaging. Magn. Reson. Med. 2003; 50: 1077–1088.

Buetefisch CM, Pirog Revill K, Haut MW, Kowalski GM, Wischnewski M, Pifer M, et al. Abnormally reduced primary motor cortex output is related to impaired hand function in chronic stroke. J. Neurophysiol. 2018: jn.00715.2017.

Devanne H, Lavoie BA, Capaday C. Input-output properties and gain changes in the human corticospinal pathway. Exp. brain Res. 1997; 114: 329–38.

Guder S, Frey BM, Backhaus W, Braass H, Timmermann JE, Gerloff C, et al. The Influence of Cortico-Cerebellar Structural Connectivity on Cortical Excitability in Chronic Stroke. Cereb. Cortex 2020; 30: 1330–1344.

Möller C, Arai N, Lücke J, Ziemann U. Hysteresis effects on the input–output curve of motor evoked potentials. Clin. Neurophysiol. 2009; 120: 1003–1008.

Pasternak O, Sochen N, Gur Y, Intrator N, Assaf Y. Free water elimination and mapping from diffusion MRI. Magn. Reson. Med. 2009; 62: 717–730.

Schulz R, Frey BM, Koch P, Zimerman M, Bönstrup M, Feldheim J, et al. Cortico-Cerebellar Structural Connectivity Is Related to Residual Motor Output in Chronic Stroke. Cereb. Cortex 2017; 27: 635–645.

Schulz R, Koch P, Zimerman M, Wessel M, Bönstrup M, Thomalla G, et al. Parietofrontal motor pathways and their association with motor function after stroke. Brain 2015; 138: 1949–1960.
